# Deterministic control of photonic de Broglie waves using coherence optics

**DOI:** 10.1038/s41598-020-69950-8

**Published:** 2020-07-30

**Authors:** Byoung S. Ham

**Affiliations:** Center for Photon Information Processing, School of Electrical Engineering and Computer Science, Gwangju Institute of Science and Technology, 123 Chumdangwagi-ro, Buk-gu, Gwangju, 61005 South Korea

**Keywords:** Quantum mechanics, Optical physics, Quantum optics

## Abstract

Photonic de Broglie waves offer a unique property of quantum mechanics satisfying the complementarity between the particle and wave natures of light, where the photonic de Broglie wavelength is inversely proportional to the number of entangled photons acting on a beam splitter. Very recently, the nonclassical feature of photon bunching has been newly interpreted using the pure wave nature of coherence optics [Sci. Rep. **10**, 7,309 (2020)], paving the road to unconditionally secured classical key distribution [Sci. Rep. **10**, 11,687 (2020)]. Here, deterministic photonic de Broglie waves are presented in a coherence regime to uncover new insights in both fundamental quantum physics and potential applications of coherence-quantum metrology.

## Introduction

The nonclassical feature of anticorrelation on a beam splitter (BS), known as Hong-Oh-Mandel dip or photon bunching, has been the heart of quantum mechanics in terms of quantum entanglement, which cannot be achieved by classical means^1–5^. Unlike most anticorrelation studies based on the statistical nature of light, a deterministic solution has been recently found in a coherence manner for a particular phase relation between two input fields impinging on a BS^6^. Owing to coherence optics with a phase control, the BS-based anticorrelation can be achieved in a simple Mach–Zehnder interferometer (MZI)^6^. One of the first proofs of MZI physics for quantum mechanics was for anticorrelation using single photons^1^. For the coherence version, unconditionally secured classical key distribution has been proposed recently^7^. Although the physics of the unconditionally secured classical key distribution is based on quantum superposition, i.e., indistinguishability in the MZI paths^7^, the key carrier is not a quantum particle but a classical coherent light compatible with current fiber-optic communications networks. As debated for several decades, fundamental questions about the quantum nature of light are still an on-going important subject in the quantum optics community^8–10^.

Here in this paper, the fundamental questions of “what is the quantum nature of light? and “what is the origin of nonclassicality?” are asked and answered in terms of photonic de Broglie waves (PBWs) in a pure coherence framework based on the wave nature of light. Due to the quantum property of linear optics such as a BS or MZI^6^, however, nonclassical light itself does not have to be excluded^1^. Thus, the present paper provides a general conceptual understanding of fundamental quantum physics as well as potential applications of coherence-quantum metrology to overcome single photon-based statistical quantum limitations such as an extremely low rate at the higher-order entangled photon-pair generation and practical difficulties of generating higher-order entangled photon pairs of NOON states^11–16^.

The photonic de Broglie wavelength ${\lambda }_{B}$ is a key feature of quantum mechanics regarding wave-particle duality and complementarity of the quantum nature of light, where classical physics is not capable of explaining such phenomena^11–16^. PBWs have been demonstrated using entangled photon pairs generated from a spontaneous parametric down conversion (SPDC) process, where ${\lambda }_{B}={\uplambda }_{0}/\mathrm{N}$, and ${\uplambda }_{0}$ (N) is the initial wavelength (number of entangled photons) of nonclassical light^11–16^. For example, a single-photon entangled pair on a beam splitter results in a PBW at ${\lambda }_{B}={\uplambda }_{0}/2$. Similarly, this is also the case for ${\lambda }_{B}={\uplambda }_{0}/4$ for a two-photon entangled pair^13^. Due to experimental difficulties of obtaining a higher-order entangled photon pair, however, the application of quantum PBWs has been severely limited so far, whose latest record is ${\lambda }_{B}={\uplambda }_{0}/18$ with N = 18^16^. By the same reasoning, quantum lithography and quantum sensing have also been limited^17–19^. In particular, no deterministic entangled photon pair generator exists.

In the present paper, deterministic control of PBWs using coherence-optics-based anticorrelation^6^ is presented for both fundamental physics and its potential applications to coherence-quantum metrology, where the order N in ${\lambda }_{B}$ is unbounded. The deterministic control of PBWs should beneficial to quantum sensors beyond the standard quantum limit. The deterministic controllability of higher-order PBWs is a breakthrough in the practical limitations of entangled photon-based conventional quantum metrology^17–19^. Most importantly, a more general understanding of the quantum nature of light is presented.

## Results

**Figure 1:**
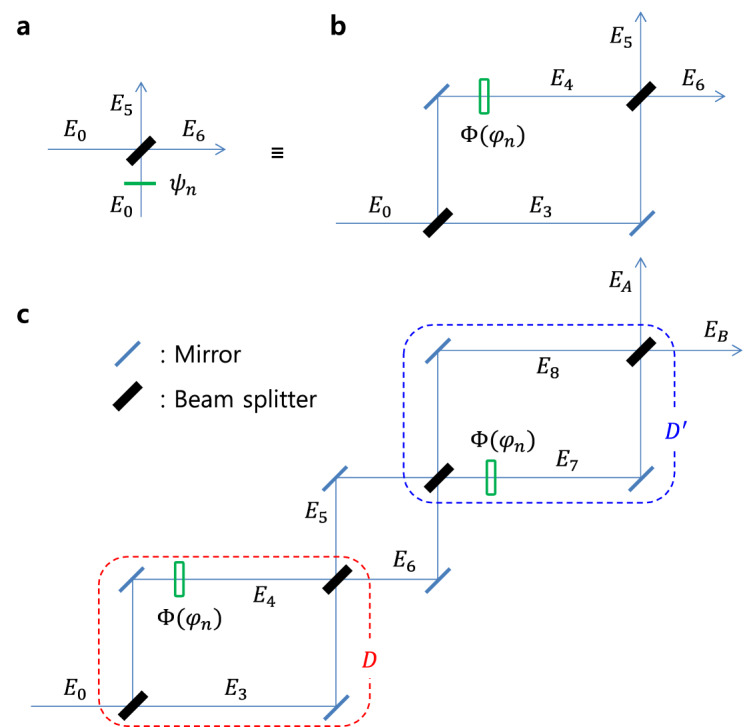
A basic unit of coherence PBW. **a** A BS-based anticorrelation scheme for photon bunching. **b** An equivalent scheme of (**a**). **c** A basic unit of coherence PBW. The input field E_0_ is coherent light. D or D’ indicates a MZI building block composed of beam splitters and a phase shifter. The coupled matrix of ${[D}{^{\prime}}][D]$ represents a coherence PBW scheme equivalent to quantum PBW with N = 4.

Figure 1 shows the basic building block of coherence PBWs using coherence optics-based anticorrelation. Figure 1a shows a deterministic scheme of anticorrelation with a phase shifter ${\psi }_{n}$ for photon bunching or a HOM dip on a BS^6^. The controlled phase ${\psi }_{n}$ is used to clarify statistical single photon-based anticorrelation^1–5^, where such anticorrelation on a BS must satisfy the definite phase between two input photons: ${\psi }_{n}=\pm \left(n-1/2\right)\pi$ for n = 1,2,3…^6^. Thus, the ambiguity in conventional anticorrelation on a BS has been thoroughly removed and applied for a deterministic nonclassical light generation. Because the BS matrix satisfies a π/2 phase shift between two split outputs, i.e., reflected and transmitted light^20^, Fig. 1a can be simply represented by a typical MZI as shown in Fig. 1b. Due to the preset π/2 phase shift on the first BS for E_3_ and E_4_ in Fig. 1b, the inserted phase shifter of ${\varphi }_{n}$ must be ${\varphi }_{n}=\pm n\pi$ for the same outputs as in Fig. 1a1, 6. The intensity correlation ${g}_{56}^{(2)}(\tau )$ between two outputs ${I}_{5}$ and ${I}_{6}$ is described by ${g}_{56}^{(2)}(\tau )=\frac{\langle {I}_{5}{\left(t+\tau \right)I}_{6}(t)\rangle }{\langle {I}_{5}(t+\tau )\rangle \langle {I}_{6}(t)\rangle }$, where ${I}_{j}$ is the intensity of ${E}_{j}$. Thus, conventional MZI becomes a quantum device for nonclassical photon generation with determinacy for Schrödinger’s cat or macroscopic NOON state generation^1^ (discussed below).

In conventional photon bunching phenomenon as exhibited in a HOM dip using SPDC-based entangled photon pairs, the requirement of $${\psi }_{n}$$ in Fig. 1a is automatically satisfied by a closed-type $${\chi }^{(2)}$$-based three-wave mixing process in a nonlinear medium. In the SPDC nonlinear optical process, however, the choice of the sign of $${\psi }_{n}$$ cannot be deterministic due to the bandwidth-wide, probabilistically distributed space-superposed entangled photons, e. g., as described by a polarization entanglement superposition state^2^: $$|\psi \rangle =\left({|H\rangle }_{1}{|V\rangle }_{2}+{{e}^{i\psi }|V\rangle }_{1}{|H\rangle }_{2}\right)/\sqrt{2}$$. In the case of two independent solid-state emitters, the generated single photon pair can be phase-locked if excited by the same pump pulse. Thus, the condition of $${\psi }_{n}$$ in Fig. 1a must be postadjusted to $$\pm \frac{\uppi }{2}$$ for the relative phase difference for anticorrelation or entangled state generation^3^. The proof of the required phase of $$\pm \frac{\uppi }{2}$$ in Fig. 1a for nonclassical light generation has already been demonstrated via two independent trapped ions^21^. In Fig. 1b, the spectral bandwidth $$(\mathrm{\delta \omega })$$ of the input light E_0_ should limit the interaction time (τ) or coherence length ($${l}_{C})$$ in $${g}_{56}^{(2)}(0)$$ anticorrelation. In the application of unconditionally secured communications^7^, the transmission distance is potentially unbounded on earth if a sub-mHz linewidth laser is used^22^: $${l}_{C}=\frac{c}{mHz}\sim {10}^{8} (km)$$. In this case, a common phase encoding technique may be advantageous compared to the amplitude modulation method. According to ref. ^23^, the maximal indistinguishability induced by perfect quantum superposition represents maximal coherence, where maximal coherence is a prerequisite for an entangled pair or anticorrelation^6^.

**Figure 2:**
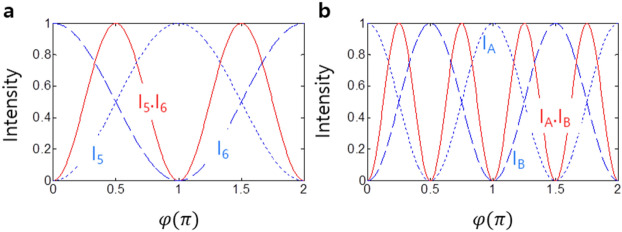
Numerical calculations for ${g}_{ij}^{(2)}(\tau )$ intensity correlation of Fig. 1(c)**.** **a** Red: ${I}_{5}{I}_{6}$ (normalized), Dotted: ${I}_{5}$, Dashed: ${I}_{6}$. **b** Red: *I*_*A*_*I*_*B*_ (normalized), Dotted: ${I}_{A}$, Dashed: *I*_*B*_,. The input field intensity of ${E}_{0}=1$ is assumed.

Figure 1c presents the basic building block of asymmetrically coupled double-MZI (ACD-MZI) for a deterministic control of PBWs via coherence optics-based anticorrelation. The output fields in the first building block D of Fig. 1c, whether it is for E_5_ or E_6_, are fed into the second block D’ by splitting them into E_7_ and E_8_, resulting in a second-order superposition state. Here, the condition (basis) of anticorrelation in the MZI is ${\varphi }_{n}=\pm n\pi$, resulting in a distinctive output of either E_5_ or E_6_. The same phase shifter is used and simultaneously controlled in both D and D’ in an asymmetric configuration (see the phase shifter $\Phi (\mathrm{\varphi })$ asymmetrically located). If the phase shifter distribution is symmetric, then a unitary transformation is satisfied for unconditionally secured classical cryptography^7^. The second-order superposition in Fig. 1c represents the fundamental physics of coherence PBWs. The output of the first block D in Fig. 1c is described as follows:

1 $$\left[ {\begin{array}{*{20}l} {E_{5} } \\ {E_{6} } \\ \end{array} } \right] = \left[ D \right]\left[ {\begin{array}{*{20}l} {E_{0} } \\ 0 \\ \end{array} } \right] = \frac{1}{2}\left[ {\begin{array}{*{20}l} {1 - e^{i\varphi } } & {i\left( {1 + e^{i\varphi } } \right)} \\ {i\left( {1 + e^{i\varphi } } \right)} & {e^{i\varphi } - 1} \\ \end{array} } \right]\left[ {\begin{array}{*{20}l} {E_{0} } \\ 0 \\ \end{array} } \right],$$

where $\left[D\right]=\left[BS\right]\left[\Phi \right]\left[BS\right]$, $$\left[BS\right]=\frac{1}{\sqrt{2}}\left[\begin{array}{cc}1& i\\ i& 1\end{array}\right]$$, and $$\left[\Phi \right]=\left[\begin{array}{cc}1& 0\\ 0& {e}^{i\varphi }\end{array}\right]$$. Here, the output fields E_5_ and E_6_ are tolerant to fluctuations in phase, frequency, and intensity of the input field E_0_. As already known in MZI interferometry, Eq. (1) shows a $2\pi$ modulation period for each output intensity: ${I}_{5}={I}_{0}\left(1-\mathrm{cos}(\varphi )\right)$; ${I}_{6}={I}_{0}\left(1+\mathrm{cos}(\varphi )\right)$ as shown in Fig. 2a. Thus, the intensity correlation ${g}_{56}^{\left(2\right)}(0)$ has a $\pi$ modulation as expected (see the red curve in Fig. 2a):

2 $$g_{56}^{\left( 2 \right)} \left( 0 \right) = \left[ {1 - {\cos}\left( {2\varphi } \right)} \right]/2,$$

where the phase basis for ${g}_{56}^{\left(2\right)}(0)=0$ is ${\varphi }_{n}=\pm n\pi$. Equation (2) provides a new understanding of nonclassical features, where perfect coherence plays a major role^6^. Equation (2) is also known as the classical resolution limit of Rayleigh criterion^24^.

The output lights, E_A_ and E_B_, in the second block D′ of Fig. 1c are then described by the following matrix relation:

3 $$\left[ {\begin{array}{*{20}l} {E_{A} } \\ {E_{B} } \\ \end{array} } \right] = \left[ {D^{\prime}} \right]\left[ D \right]\left[ {\begin{array}{*{20}l} {E_{0} } \\ 0 \\ \end{array} } \right] = - \frac{1}{2}\left[ {\begin{array}{*{20}l} {1 + e^{i2\varphi } } & {i\left( {1 - e^{i2\varphi } } \right)} \\ { - i\left( {1 - e^{i2\varphi } } \right)} & {1 + e^{i2\varphi } } \\ \end{array} } \right]\left[ {\begin{array}{*{20}l} {E_{0} } \\ 0 \\ \end{array} } \right],$$

where $\left[D{^{\prime}}\right]=\left[BS\right]\left[{\Phi }{^{\prime}}\right][BS]$ and $$\left[{\Phi }{^{\prime}}\right]=\left[\begin{array}{cc}{e}^{i\varphi }& 0\\ 0& 1\end{array}\right]$$. Unlike Eqs. (1), (3) results in a twice shorter (faster) modulation period (frequency), i.e., a π modulation in each intensity of I_A_ and I_B_: ${I}_{A}=\frac{1}{2}\left(1+\mathrm{cos}\left(2\mathrm{\varphi }\right)\right)$; ${I}_{B}=\frac{1}{2}\left(1-\mathrm{cos}\left(2\mathrm{\varphi }\right)\right)$ (see Fig. 2b). As a result, the intensity correlation ${g}_{AB}^{\left(2\right)}(0)$ of I_A_ and I_B_ becomes:

4 $$g_{AB}^{\left( 2 \right)} \left( 0 \right) = \left[ {1 - {\cos}\left( {4\varphi } \right)} \right]/2$$

Thus, it has proved that the classical resolution limit of ${\lambda }_{0}/2$ governed by the Rayleigh criterion in Fig. 2a is overcome using coherence optics in the ACD-MZI scheme of Fig. 1c. This is the first analytic proof of such nonclassical features obtained by pure coherence optics. In Eq. (4), the phase basis for ${g}_{AB}^{\left(2\right)}(0)=0$ is accordingly changed from ${\varphi }_{n}=\pm n\pi$ in Fig. 2a to ${\varphi }_{n}=\pm n\pi /2$ in Fig. 2b. This doubly enhanced resolution of the output intensity in Eq. (4) contradicts our general understating on quantum mechanics because the method presented in Fig. 1c is perfectly coherent and classical.

**Figure 3:**
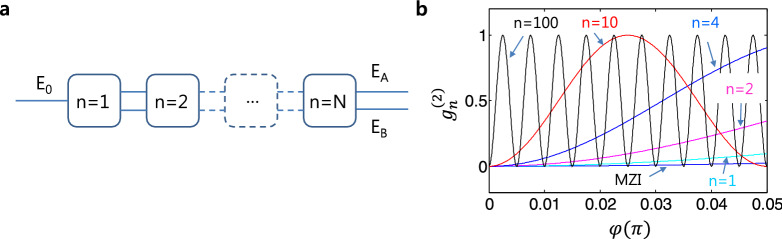
A photonic de Broglie wavelength generator. **a** A serially connected ACD-MZI. Each block represents ACD-MZI of Fig. 1c. **b** Numerical calculations for (**a)**, where n indicates the number of blocks in **a**. MZI represents a reference of a classical limit whose period is $\uppi$ as shown in Fig. 2a.

Here, it should be noted that perfect correlation between two lights (E_3_/E_4_ or E_7_/E_8_) is achieved by path indistinguishability in MZI, and proved by either anticorrelation^1–6^ or Bell inequality violation^25–28^. Thus, the specific phase relation with ${\varphi }_{n}$ between two superposed coherent lights in MZI of Fig. 1b,c is the source of nonclassical features such as anticorrelation and entanglement^6^. In that sense, the number of superposition states in Fig. 1c should be equivalent to the number of entangled photons in conventional quantum PBWs (see Eqs. 2 and 4). Therefore, the ACD-MZI scheme of Fig. 1c represents the basic unit of the present coherence model of PBWs. As a result, higher-order coherence PBWs can be generated by simply connecting the asymmetrical units of Fig. 1c in a series (discussed in Fig. 3).

Figure 2 shows numerical calculations for Fig. 1c to support the present theory of deterministic control of PBWs in a coherence regime. Figure 2a shows a typical MZI result of Fig. 1b by solving Eq. (1), where each output intensity represents the classical limit. As expected, the conventional MZI scheme has a spectroscopic resolution of ${\uplambda }_{0}/2$, which is the Rayleigh limit in classical physics. This classical resolution limit is now understood as the first order of the present ACD-MZI PBWs: ${\lambda }_{CB}={\uplambda }_{0}/2\zeta$, where $\upzeta$ is the number of MZI blocks (or superposition states in the form of Fig. 1b), and ${\lambda }_{CB}$ indicates the present coherence PBW. Here, it should be noted that each MZI block in Fig. 1c is equivalent to N = 2 in a quantum PBW^11–16^ for an entanglement superposition description of $|\psi \rangle =\left({|N\rangle }_{A}{|0\rangle }_{B}+{|0\rangle }_{A}{|N\rangle }_{B}\right)/\sqrt{2}$: $2\upzeta =\mathrm{N}$. In other words, a typical MZI is a quantum device for anticorrelation or nonclassical light generation if ${\varphi }_{n}=\pm n\pi$ is satisfied. The intensity correlation of ${g}_{AB}^{\left(2\right)}(0)$ in Eq. (4) is numerically calculated in Fig. 2b (see the red curve). The demonstration of ${\lambda }_{CB}={\uplambda }_{0}/4$ in Fig. 2b validates the present theory of coherence PBWs based on Fig. 1c. Thus, it is concluded that the present coherence PBW in Fig. 1c is equivalent to the quantum PBW^11–16^ based on entangled photons with the additional benefit of deterministic controllability.

For the higher order ${\lambda }_{CB}$, the basic scheme of Fig. 1c needs to be repeated in a row as shown in Fig. 3a. In a serial connection, the output from each block becomes two inputs for the next block without loss. Defining $\left[CM\right]=\left[{D}{^{\prime}}\right]\left[D\right]$ with no loss, the nth output fields in Fig. 3a can be obtained from Eq. (3) as (see the Supplementary information):

5-1 $$\left[ {\begin{array}{*{20}l} {E_{A} } \\ {E_{B} } \\ \end{array} } \right]^{n} = \left[ {CM} \right]^{n} \left[ {\begin{array}{*{20}l} {E_{0} } \\ 0 \\ \end{array} } \right],$$

5-2 $$= \frac{1}{2}\left( { - 1} \right)^{n} \left[ {\begin{array}{*{20}l} {\left( {1 + e^{i2n\varphi } } \right)} & {i\left( {1 - e^{i2n\varphi } } \right)} \\ { - i\left( {1 - e^{i2n\varphi } } \right)} & {\left( {1 + e^{i2n\varphi } } \right)} \\ \end{array} } \right]\left[ {\begin{array}{*{20}l} {E_{0} } \\ 0 \\ \end{array} } \right],$$

5-3 $$\left( {E_{A} } \right)^{n} = \frac{{E_{0} }}{2}\left( { - 1} \right)^{n} \left( {1 + e^{i2n\varphi } } \right),$$

5-4 $$\left( {E_{B} } \right)^{n} = i\frac{{E_{0} }}{2}\left( { - 1} \right)^{n + 1} \left( {1 - e^{i2n\varphi } } \right).$$

From Eqs. (5–3) and (5–4) the related nth output intensities are obtained:

6-1 $$\left( {I_{A} } \right)^{n} = \frac{1}{2}I_{0} \left[ {1 + {\cos}\left( {2n\varphi } \right)} \right],$$

6-2 $$\left( {I_{B} } \right)^{n} = \frac{1}{2}I_{0} \left[ {1 - {\cos}\left( {2n\varphi } \right)} \right].$$

where ${I}_{0}={E}_{0}{E}_{0}^{*}$. Regardless of the chain length, the final output intensity is always the same as the input if optical loss is neglected. As a result, the intensity correlation ${g}_{n}^{\left(2\right)}(0)$ becomes:

7 $$g_{n}^{\left( 2 \right)} \left( 0 \right) = \frac{{\left( {I_{A} } \right)^{n} \left( {I_{B} } \right)^{n} }}{{\left( {I_{A} } \right)^{n} \left( {I_{B} } \right)^{n} }} = \frac{1}{2}\left[ {1 - {\cos}\left( {4n\varphi } \right)} \right],$$

where ${g}_{n}^{\left(2\right)}\left(0\right)$ represents for ${g}_{AB}^{\left(2\right)}(0)$ of the nth output in Fig. 3a. Thus, the general solution for the nth coherence PBW in Fig. 3a is:

8 $$\lambda_{CB}^{\left( n \right)} = \lambda_{0} /4n,$$

where n is the number of ACD-MZI of Fig. 1c. For n = 1, it is equivalent to the four-photon (N = 4) case in quantum PBW^11–16^. Because Eq. (8) is deterministic, the coherence PBW is powerful compared with its conventional quantum counterpart in terms of N in the determinacy, controllability, and intensity. These facts may open the door to coherence-quantum metrology based on on-demand ${\lambda }_{CB}^{(n)}$. Compared with the impractical quantum PBWs, where it takes ~ 2 h of acquisition time just for N = 18^16^, the present coherence PBWs is unbounded in both N and power, and real time in processing due to coherence optics.

Figure 3a represents a serially connected ACD-MZI scheme, where each block is equivalent to Fig. 1c as a four-photon quantum PBW: ${\lambda }_{B} (=\frac{{\lambda }_{0}}{4})$. Regarding the connection lines, only one line is active for coherence PBWs, where the anticorrelation condition ${\varphi }_{n}=\pm n\pi$ is satisfied. If an error is found in the output intensity, that means the maximum superposition in MZI is broken and both lines are active. The error sharpness is the spectroscopic resolution of the coherence PBW for coherence-quantum metrology. To realize the schematic of Fig. 3a, a waveguide-coupled^29^ or a fiber-coupled^30^ MZI scheme would be a good example (see the Supplementary Information).

Figure 3b shows numerical calculations using Eq. (7) for the intensity correlation ${g}_{n}^{\left(2\right)}(0)$. As shown in Fig. 3b, the coherence ${\lambda }_{CB}^{(n)}$ is equivalent to the quantum ${\lambda }_{B}$. Compared with a quantum PBW^11–16^, the coherence PBW at ${\lambda }_{CB}^{(n)}$ is deterministic, macroscopic, functioning in real time, and boundless on N. Each intensity modulation period for ${\left({I}_{A}\right)}^{n}$ or ${\left({I}_{B}\right)}^{n}$ is twice as long as ${g}_{n}^{\left(2\right)}(0)$, as shown in Eqs. (6–1) and (6–2).

## Conclusion

In conclusion, the deterministic control of photonic de Broglie waves (PBWs) was presented based solely on coherence optics for both fundamental physics and potential applications of quantum metrology using a chain of ACD-MZIs. For this, the output from each ACD-MZI was directly inserted into the next one until reaching the given n, where n indicates the number of ACD-MZIs. The analytical expressions and their numerical calculations successfully demonstrated the nonclassical features equivalent to quantum PBWs, where the number of MZIs in the coherence PBWs is equivalent to the entangled photon number N in quantum PBWs. The random phase noise of the MZI system caused by mechanical vibrations, air turbulence, and temperature variations at $\le$ MHz may be eliminated or minimized through the use of either silicon photonics or fiber-optic technologies. Both stability and linewidth of the input light should act as the bound of coherence PBWs, limiting MZI fringe resolution. Thus, a fine-tuned laser such as sub-mHz laser should provide a higher n for shorter PBWs^31^. As a result, the proposed coherence PBW can be directly applied to high-precision optical spectroscopy or quantum metrology such as optical clocks^32^, gravitational wave detection^33^, quantum lithography^17,18^, and quantum sensors^19^. The seemingly contradictory aspect of coherence PBWs with quantum physics is reconciled via the quantum superposition of MZI paths, where MZI is treated as a quantum device like a BS if the appropriate phase is satisfied^1,6^. Thus, the present scheme of Fig. 3a may open the door to coherence-quantum metrology for deterministic control of photonic de Broglie wavelengths at higher orders in real-time and on-demand settings. Eventually, the present coherence photonic de Broglie wave generation scheme may be applied to non-classical light generation, such as deterministic entangled photons and photonic qubits, resulting in on-demand quantum information processing in a macroscopic regime.

## Methods

The numerical calculations in Figs. 2 and 3 were performed by Matlab using equations appeared in the text. The data that support the findings of this study are available from the corresponding author upon reasonable request.

## Supplementary information

Supplementary information.
