# Supplementary material for: Deterministic control of photonic de Broglie waves using coherence optics
Source: Sci Rep. 2020 Jul 30;10:12899. doi: 10.1038/s41598-020-69950-8 (PMC7393373; doi:10.1038/s41598-020-69950-8)
Supplement: Supplementary file 1 — Supplementary information. [file 41598_2020_69950_MOESM1_ESM.pdf]

Supplementary Information for

Deterministic control of photonic de Broglie waves using coherence optics: Coherence de Broglie waves,

by BS Ham

A. Analysis for Eqs. (6-1) and (6-2)

Assuming there is an optical loss in each block in Fig. 3(a),

$$\begin{bmatrix} E_A \\ E_B \end{bmatrix}^1 = [CM]^1 \eta^1 \begin{bmatrix} E_0 \\ 0 \end{bmatrix},$$
$$= (-1)^1 \left(\frac{1}{2}\right)^1 \eta^1 \begin{bmatrix} 1+e^{i2\varphi} & i(1-e^{i2\varphi}) \\ -i(1-e^{i2\varphi}) & 1+e^{i2\varphi} \end{bmatrix} \begin{bmatrix} E_0 \\ 0 \end{bmatrix}, \quad \text{(S1)}$$

where the superscript indicates the number of the basic building block of Fig. 1(c), and η is transmittance T.
For $\varphi = \pm m\pi$, thus, $E_A = -\eta E_0$ and $E_B = 0$, where, m=0,1,2,3…
For $\varphi = \pm\frac{(2m+1)}{2}\pi$, $E_A = 0$ and $E_B = i\eta E_0$. For n=2 in Fig. 3(a),

$$\begin{bmatrix} E_A \\ E_B \end{bmatrix}^2 = [CM]^2 \begin{bmatrix} E_0 \\ 0 \end{bmatrix},$$
$$= [CM][CM]\eta^2 \begin{bmatrix} E_0 \\ 0 \end{bmatrix},$$
$$= (-1)^2 \left(\frac{1}{2}\right)^2 \eta^2 \begin{bmatrix} 1+e^{i2\varphi} & i(1-e^{i2\varphi}) \\ -i(1-e^{i2\varphi}) & 1+e^{i2\varphi} \end{bmatrix} \begin{bmatrix} 1+e^{i2\varphi} & i(1-e^{i2\varphi}) \\ -i(1-e^{i2\varphi}) & 1+e^{i2\varphi} \end{bmatrix} \begin{bmatrix} E_0 \\ 0 \end{bmatrix},$$
$$= (-1)^2 \left(\frac{1}{2}\right)^2 \eta^2 \begin{bmatrix} (1+e^{i2\varphi})^2 + (1-e^{i2\varphi})^2 & 2i(1+e^{i2\varphi})(1-e^{i2\varphi}) \\ -2i(1+e^{i2\varphi})(1-e^{i2\varphi}) & (1+e^{i2\varphi})^2 + (1-e^{i2\varphi})^2 \end{bmatrix} \begin{bmatrix} E_0 \\ 0 \end{bmatrix},$$
$$= \frac{1}{2}(-1)^2\eta^2 \begin{bmatrix} (1+e^{i4\varphi}) & i(1-e^{i4\varphi}) \\ -i(1-e^{i4\varphi}) & (1+e^{i4\varphi}) \end{bmatrix} \begin{bmatrix} E_0 \\ 0 \end{bmatrix}. \quad \text{(S2)}$$

For $\varphi = \pm m\pi/2$, $(E_A)^{n=2} = \eta^2 E_0$ and $E_B = 0$.
For $\varphi = \pm\frac{(2m+1)}{4}\pi$, $(E_A)^{n=2} = 0$ and $E_B = -i\eta^2 E_0$. For n=3 in Fig. 3(a),

$$\begin{bmatrix} E_A \\ E_B \end{bmatrix}^3 = [CM]^3 \begin{bmatrix} E_0 \\ 0 \end{bmatrix},$$
$$= [CM][CM][CM]\eta^3 \begin{bmatrix} E_0 \\ 0 \end{bmatrix},$$
$$= (-1)^3 \left(\frac{1}{2}\right)^3 2^1\eta^3 \begin{bmatrix} (1+e^{i2\varphi}) & i(1-e^{i2\varphi}) \\ -i(1-e^{i2\varphi}) & (1+e^{i2\varphi}) \end{bmatrix} \begin{bmatrix} (1+e^{i4\varphi}) & i(1-e^{i4\varphi}) \\ -i(1-e^{i4\varphi}) & (1+e^{i4\varphi}) \end{bmatrix} \begin{bmatrix} E_0 \\ 0 \end{bmatrix},$$
$$= \frac{1}{2}(-1)^3\eta^3 \begin{bmatrix} (1+e^{i6\varphi}) & i(1-e^{i6\varphi}) \\ -i(1-e^{i6\varphi}) & (1+e^{i6\varphi}) \end{bmatrix} \begin{bmatrix} E_0 \\ 0 \end{bmatrix}. \quad \text{(S3)}$$

For $\varphi = \pm m\pi/3$, $(E_A)^{n=2} = -\eta^3 E_0$ and $E_B = 0$.
For $\varphi = \pm\frac{(2m+1)}{6}\pi$, $(E_A)^{n=2} = 0$ and $E_B = i\eta^3 E_0$. For n=4 in Fig. 3(a),

$$\begin{bmatrix} E_A \\ E_B \end{bmatrix}^4 = [CM]^4\eta^4 \begin{bmatrix} E_0 \\ 0 \end{bmatrix},$$
$$= (-1)^4 \left(\frac{1}{2}\right)^4 2^2\eta^4 \begin{bmatrix} (1+e^{i2\varphi}) & i(1-e^{i2\varphi}) \\ -i(1-e^{i2\varphi}) & (1+e^{i2\varphi}) \end{bmatrix} 2^2 \begin{bmatrix} (1+e^{i6\varphi}) & i(1-e^{i6\varphi}) \\ -i(1-e^{i6\varphi}) & (1+e^{i6\varphi}) \end{bmatrix} \begin{bmatrix} E_0 \\ 0 \end{bmatrix},$$
$$= \frac{1}{2}(-1)^4\eta^4 \begin{bmatrix} (1+e^{i8\varphi}) & i(1-e^{i8\varphi}) \\ -i(1-e^{i8\varphi}) & (1+e^{i8\varphi}) \end{bmatrix} \begin{bmatrix} E_0 \\ 0 \end{bmatrix}. \quad \text{(S4)}$$

For $\varphi = \pm m\pi/4$, $(E_A)^{n=2} = \eta^4 E_0$ and $E_B = 0$.

For $\varphi = \pm\frac{(2m+1)}{8}\pi$, $(E_A)^{n=2} = 0$ and $E_B = -i\eta^4 E_0$.

From the above relations, the following nth outputs can be driven as a general relation:

$$\begin{bmatrix} E_A \\ E_B \end{bmatrix}^n = [CM]^n \eta^n \begin{bmatrix} E_0 \\ 0 \end{bmatrix},$$
$$= \frac{1}{2}(-1)^n \eta^n \begin{bmatrix} (1+e^{i2n\varphi}) & i(1-e^{i2n\varphi}) \\ -i(1-e^{i2n\varphi}) & (1+e^{i2n\varphi}) \end{bmatrix} \begin{bmatrix} E_0 \\ 0 \end{bmatrix}. \quad \text{(S5)}$$

For $\varphi = \pm\left(\frac{m}{n}\right)\pi$, $(E_A)^n = (-1)^n \eta^n E_0$ and $E_B = 0$.

For $\varphi = \pm\left(\frac{(2m+1)}{2n}\right)\pi$, $(E_A)^n = 0$ and $E_B = i(-1)^{n+1}\eta^n E_0$. As a result,

$$(I_A)^n = \frac{1}{4}\eta^{2n} I_0 (1+e^{i2n\varphi})(1+e^{-i2n\varphi}) = \frac{1}{2}\eta^{2n} I_0 [1+\cos(2n\varphi)], \quad \text{(S6)}$$

where $I_0 = E_0 E_0^*$. Likewise,

$$(I_B)^n = \frac{1}{4}\eta^{2n} I_0 (1-e^{i2n\varphi})(1-e^{-i2n\varphi}) = \frac{1}{2}\eta^{2n} I_0 [1-\cos(2n\varphi)]. \quad \text{(S7)}$$

Thus, the intensity correlation $g_n^{(2)}$ between the nth outputs I_A and I_B becomes:

$$g_n^{(2)} = \frac{\langle (I_A)^n (I_B)^n \rangle}{\langle (I_A)^n \rangle \langle (I_B)^n \rangle} = 1 - cos^2(2n\varphi) = \frac{1}{2}[1-\cos(4n\varphi)]. \quad \text{(S8)}$$

B. Analysis for ACD-MZI of coherence PBW

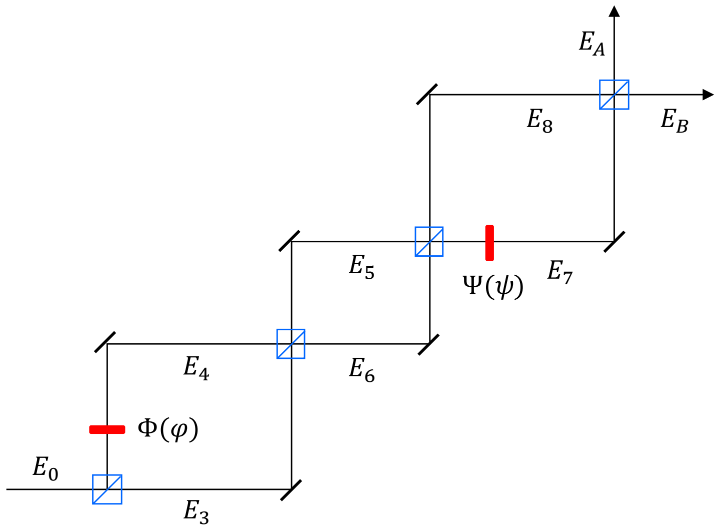

Fig. S1. A schematic of coherence PBW. The square box represents a nonpolarized beam splitter.

Figure S1 and S2 are for the general case of ACD-MZI coherence PBW in Fig. 3 for n=3, where two phase shifters behave independently.

Figure S2 is numerical calculations of Fig. S1 for each output field. Figure S2(c) is for Figs. S2(a) (blue) and S2(b) (red) along the off-diagonal direction with $\varphi = \psi$, where the modulation period of each output intensity is $2\pi/3$ as expected. For $\varphi = -\psi$, it is along the diagonal direction in Fig. S2(a) and S2(b). Specifically for

φ = −ψ = π, I_A represents a constant I_0 along the red line, which is identity relation[7]. So does I_B along the blue line represents for –I_0. This is the case of time reversal process in an optical (quantum) memory.

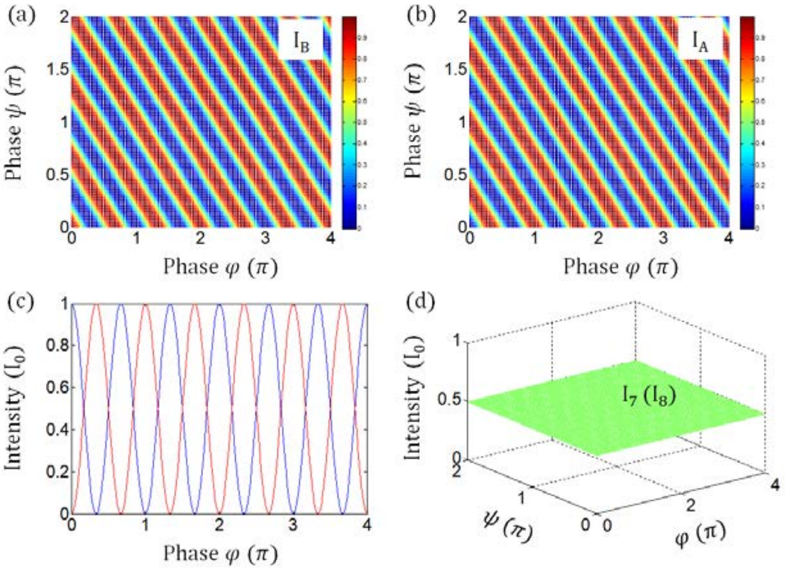

Fig. S2. Numerical calculations for Eqs. (S6) and (S7) for n=3. (a) Intensity of output E_B: I_B, (b) Intensity of output E_A: I_A, (c) I_A (Red); I_B (Blue), and (d) Intensity of output E_7: I_7 (=I_8): $I_0 = E_0 E_0^*$.

Figure S3 shows a schematic diagram for the potential application of the present coherence PBW with n=36 in Eq. (8) using, e.g., a silicon-based waveguide structure. Owing to the high precision nano-fabrication technologies with a temperature controller (TEC) in each waveguide, the phase change in the MZI can be easily controlled. The resulting PBW is $\lambda_{CB} = \lambda_0/144$.

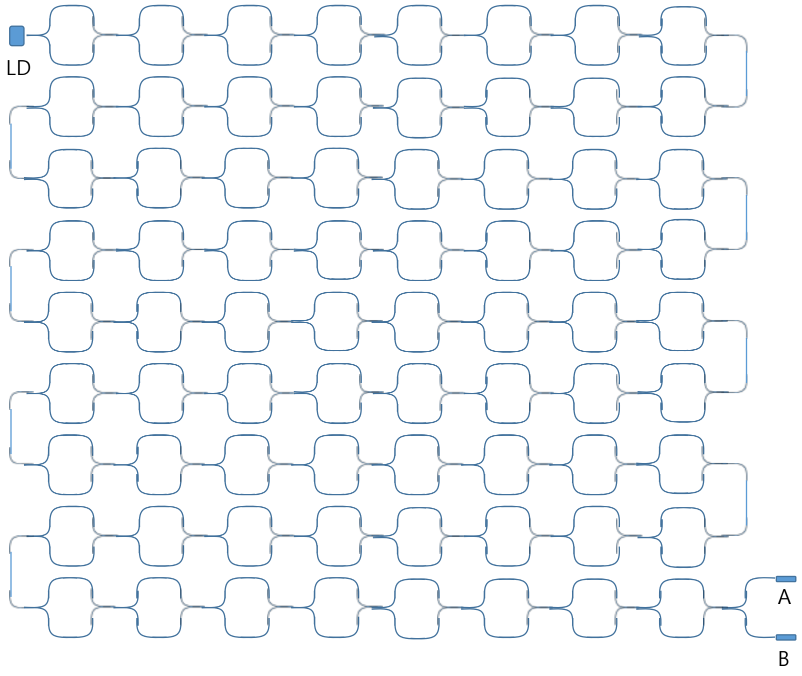

Fig. S3. A schematic of serial connection of ACD-MZI for coherence PBW at $\lambda_{CB}^{(n=36)}$.
